# Surface-Bound Molecular Gradients for the High-Throughput Screening of Cell Responses

**DOI:** 10.3389/fbioe.2015.00132

**Published:** 2015-08-31

**Authors:** Anna Lagunas, Elena Martínez, Josep Samitier

**Affiliations:** ^1^Networking Biomedical Research Center in Bioengineering, Biomaterials and Nanomedicine (CIBER-BBN), Madrid, Spain; ^2^Nanobioengineering Group, Institute for Bioengineering of Catalonia (IBEC), Barcelona, Spain; ^3^Biomimetic Systems for Cell Engineering Group, Institute for Bioengineering of Catalonia (IBEC), Barcelona, Spain; ^4^Department of Electronics, University of Barcelona (UB), Barcelona, Spain

**Keywords:** molecular gradient, cell adhesion, cell morphology, cell growth, cell differentiation

## Abstract

Chemical gradient surfaces are described as surfaces with a gradually varying composition along their length. Continuous chemical gradients have recently been proposed as an alternative to discrete microarrays for the high-throughput screening of the effects of ligand concentration in cells. Here, we review some of the most recent examples in which gradients have been used to evaluate the effect of a varying ligand concentration in cell adhesion, morphology, growth, and differentiation of cells, including some of our recent findings. They show the importance of the organization of ligands at the nanoscale, which is highlighted by abrupt changes in cell behavior at critical concentration thresholds.

## Introduction

During embryo development, cell patterning is governed by underlying morphogen gradients. The idea of a morphogen gradient is intimately associated with the concept of positional information: a cell can read its position in the gradient and respond accordingly (Gurdon and Bourillot, 2001). This idea is not only applicable to cell differentiation but also to many other cell processes. Cells can recognize different threshold concentrations of signaling molecules through receptors in their surface and transduce this information to the nucleus for the appropriate cell response. Therefore, control of ligand dosage is critical in the evaluation of ligand effects on cells.

Since many signaling molecules, such as growth factors, can function under restricted diffusion conditions, surface confinement does not compromise the biological relevance of surface-bound ligand dosage assays. For the systematic *in vitro* study of ligand concentration on cell signaling, the microarray format is commonly used. Cell microarrays allow for the high-throughput screening of the effects of signaling molecules printed alone or in combination, and significantly reduce the amount of reagents needed and the inter-experimental variability of conventional microwell plate tests (Miller et al., 2006; Rodríguez-Seguí et al., 2011; Papp et al., 2012; Warmflash et al., 2014). However, even if a large number of ligand concentrations can be included in a microarray, these are inherently discrete.

Since *in vivo* cells respond to small changes in tiny amounts of signaling molecules, a more accurate screening could be provided by continuous chemical gradients. Chemical gradients may be affected by some physical cues, such as changes in stiffness or topography along the gradient distance, that can influence cell behavior and cause a biasing of the inferred results. Picart and co-workers show how an increase of stiffness from 200 to 600 kPa (slope 9.90 kPa/mm) in polyelectrolyte multilayer (PEM)-based gradients caused an increase of adhesion and spreading (cell area varied between 500 and 2500 μm^2^ with increasing stiffness) of the MC3T3-E1 pre-osteoblastic cells (Almodóvar et al., 2013). Moreover, some recent works examined the interplay between substrates stiffness and cell-adhesive coatings in the mechanical feedback received by the exposed cells, affecting stem cell fate (Trappmann et al., 2012; Wen et al., 2014). Gadegaard and co-workers observed that hTERT fibroblast cell line aligns and polarizes in the direction of polycarbonate microgrooves in a topographical gradient in which groove pitch and depth are orthogonal and continuously varied (Reynolds et al., 2012). In that sense, it is mandatory to keep relevant parameters, such as stiffness and topography, which influence cell response, invariable along chemical gradient distance to unequivocally attribute cell responses to the introduced variations in ligand concentration. In this review, we present several examples of continuous chemical gradients, produced by different methodologies that allow for the screening of the effects of ligand concentration and the evaluation of different aspects of cell behavior, such as adhesion, morphology, and fate, are considered.

## Changes in Cell Adhesion and Morphology Introduced by Gradients

**Figure 1:**
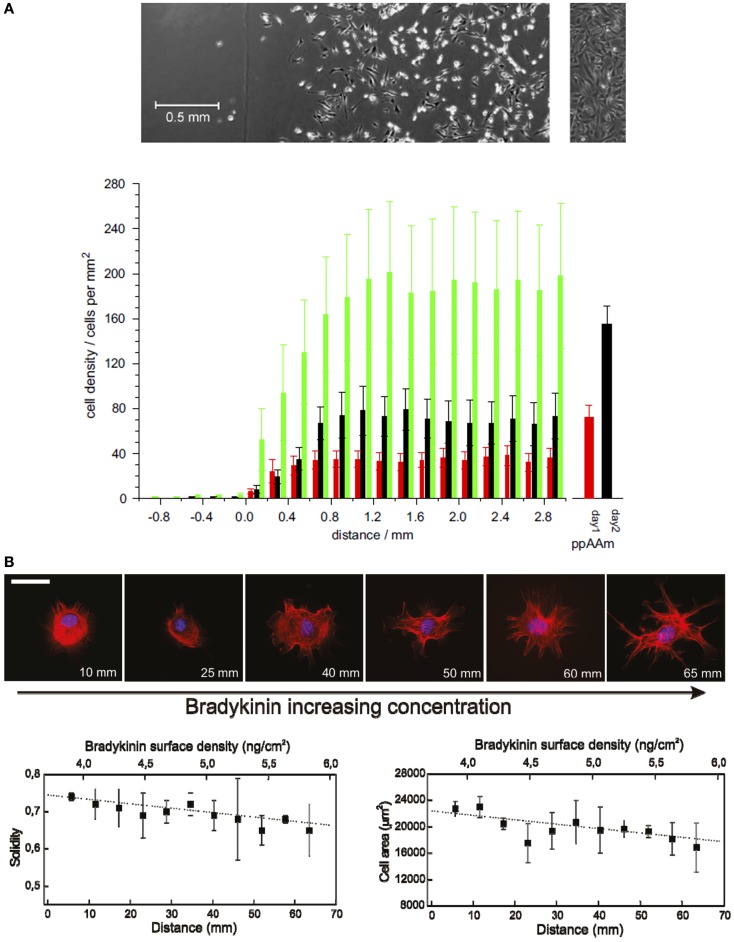
**Cell adhesion and morphology changes on continuous chemical gradients**. **(A)** Average number of cells in 0.2 mm increments along the wettability gradient (left: ppHex; right: ppAAm) after 1 (red), 2 (black), and 3 (green) days of incubation. Error bars represent SEM; *n* = 9. Sample/mask interface was set at the origin of the *x*-axis. The columns to the right are the average cell numbers on the uniform ppAAm samples after 1 and 2 days (*n* = 35). The top images show the typical cell response after 2 days on the gradient (the vertical line marks the start of the gradient) and the uniform sample. **(B)** Representative fluorescence microscopy images show the effect of BK concentration gradient on cell morphology in NIH/3T3 fibroblasts [stained for cell nuclei (blue) and actin filaments (red)] after 90 min from cell seeding. Scale bar = 50 μm. Cell membrane constrictions and filopodia formation are more evident with increasing BK concentration. Graphs below show cell solidity (left) and cell area variation (right) as a function of distance showing a progressive decrease with increasing BK concentration [**(A)**: Zelzer et al., 2008; **(B)**: Lagunas et al., 2010].

One of the most common techniques to create chemical gradients is plasma polymerization (Wittle et al., 2003). Plasma polymers provide smooth coatings that can be deposited onto any surface without changing its topography and therefore, their effects on cell response can be attributed solely to the changes produced in the surface chemistry. Alexander and co-workers produced wettability gradients by varying the surface chemical composition using a diffusion-controlled plasma polymerization technique. Gradients from the chemistry of plasma polymerized allylamine (pAAm) to that of plasma polymerized hexane (ppHex) were formed on a glass slide using diffusion under a fixed mask. A variation of the water contact angle from 94°(on the ppHex side) to 67°(on the pAA side) caused an increase of NIH 3T3 fibroblast cell density from nearly 0 to 40 cells/mm^2^ after 24 h of culture (Figure 1A; Zelzer et al., 2008). Plasma polymer gradients of acrylic acid and diethlylene glycol have been used to screen stem cell–surface interactions, showing striking differences in the size and the morphology of colonies formed by mouse embryonic stem cells along the gradient (Harding et al., 2012). In a different approach, continuous chemical gradients can be created by using surface coatings, such as self-assembled monolayers (SAMs). Mrksich and co-workers reported a method that combines gradients of soluble Arg-Gly-Asp (RGD) cell-adhesive peptide ligands in microfluidic networks with immobilization chemistries of maleimide groups on SAMs. This strategy was used to present defined gradients to individual cells and showed that the gradient of the ligand leads to a non-uniform distribution of the cytoskeleton in adhered cells (Petty et al., 2007). Yeo and co-workers (Lee et al., 2013) described the generation of multicomponent gradient surfaces based on SAMs terminated with a quinone derivative. The quinone group was progressively reduced by a linear-dipping exposure to a reducing agent, leading to a continuous gradient of amino groups that can be further reacted with extracellular matrix (ECM) ligands. They prepared RGD/Pro-His-Ser-Arg-Asn (PHSRN) gradient surfaces with various total ligand densities and observed that PHSRN enhances cell adhesion at positions where the two ligands are presented in equal amounts, while these peptide ligands competed in cell adhesion at other positions.

In our group, we developed a universal platform to create chemical gradients based on the biotin–streptavidin interaction. Gradients were generated in this case by the progressive alkaline hydrolysis of poly(methyl methacrylate) (PMMA) spin-coated onto a microscope glass slide. The carboxylate groups were then modified with biotin and finally with streptavidin. This procedure allowed obtaining low-slope gradients (0.9 pmol/cm^3^ of streptavidin) in which the surface physical properties remained almost invariable all over the gradient length (roughness RMS values were below 0.4 nm along the slide distance). In a proof of concept application, we modified the streptavidin gradients with the biotinylated bradykinin (BK) peptide. BK caused membrane ruffling and filopodia formation in NIH 3T3 fibroblasts cultured on the gradient surfaces in a concentration-dependent manner (Figure 1B; Lagunas et al., 2010). Low-slope gradients result in minute variations of concentration at distances comparable to cell size, making them very useful for the screening of cell–surface interactions. Constantino and co-workers used laser-assisted protein adsorption by photobleaching (LAPAP) for the fabrication of large-scale substrate-bound gradients of the ECM protein laminin-1 to study the reshaping process of neurite extension. They observed that low-slope gradients (with a 4.6% absolute laminin-1 concentration change along the cell diameter) were enough to produce a statistically significant guidance in neurite extension 3 h after differentiation (Bélisle et al., 2012).

With the aim of creating ECM protein gradients for cell adhesion studies, we modified our biotin–streptavidin-based platform with biotinylated RGD. We obtained linear gradients with a variation in RGD surface density that goes from 2.8 to 4.4 pmol/cm^2^. Such a low-slope gradient allowed for the identification of a threshold value of 4.0 pmol/cm^2^ for successful cell attachment and spreading of NIH 3T3 fibroblasts. We attributed this non-linear cellular response to the linear variation of RGD concentration in the gradient, to the non-homogeneous RGD surface distribution at the nanometer scale (Lagunas et al., 2012). In fact, cell adhesion process is governed mainly by the physiological arrangement of ECM at the nanoscale being more affected by local than by global ligand concentrations (Malmström et al., 2010; Deeg et al., 2011): Spatz and co-workers first developed gold nanoparticle density gradients based on block copolymer micelle nanolithography to present a molecularly controlled spacing of RGD at the nanometer scale along the gradient length. With this method, they particularly address cell response to surface presentation of individual adhesion molecules showing that cells respond to the weak slope of Δ15 nm/mm sensing spatial variations of <1 nm across the cell diameter (Arnold et al., 2008).

## Changes in Cell Differentiation and Growth Introduced by Gradients

The density of chemical functional groups plays a crucial role in affecting cellular behavior, such as growth and differentiation (Gurdon and Bourillot, 2001; Schwab et al., 2015). Continuous gradients of surface-bound molecular ligands provide an unmatched set-up for the high-throughput screening of cell responses to matrix-bound proteins and mimic cell–cell interactions. Therefore, gradients are used as a tool to establish dose–response curves of different cell types to specific extracellular matrix proteins, growth factors, and cytokines. They allow for determining in an efficient way the optimal concentration of matrix-bound biomolecules and for studying the presentation of growth factors in a spatially controlled manner.

In a pioneering work, Miller and co-workers used inkjet printing to create a non-continuous gradient of biomolecules composed by spots of increasing concentrations (Miller et al., 2009). They used this approach to print bone morphogenetic protein-2 (BMP-2) and insulin-like growth factor-II (IGF-II) on fibrin-coated substrates. They created almost linear gradients with a slope of 0.25 μg/cm^3^ (9.6 pmol/cm^3^) over 1.5 mm, which were used to determine the effect of IGF-II and BMP-2 surface concentrations on guiding C2C12 cells toward an osteogenic lineage. This proved the potential of surface gradients as high-throughput screening technique.

In a recent study, surface chemistry gradients have proved to influence the growth and differentiation of rat mesenchymal stem cells (rMSCs). Results showed that, even if the effects of the surface chemistry on cell adhesion were pronounced at an early culture stage, they can be diminished during long-term culture (Wang et al., 2015). Cell differentiation toward osteogenic or adipogenic fates was influenced by the surface chemistry gradient mainly through its influence in the cell density, which is an effect much more pronounced on the osteogenic commitment.

**Figure 2:**
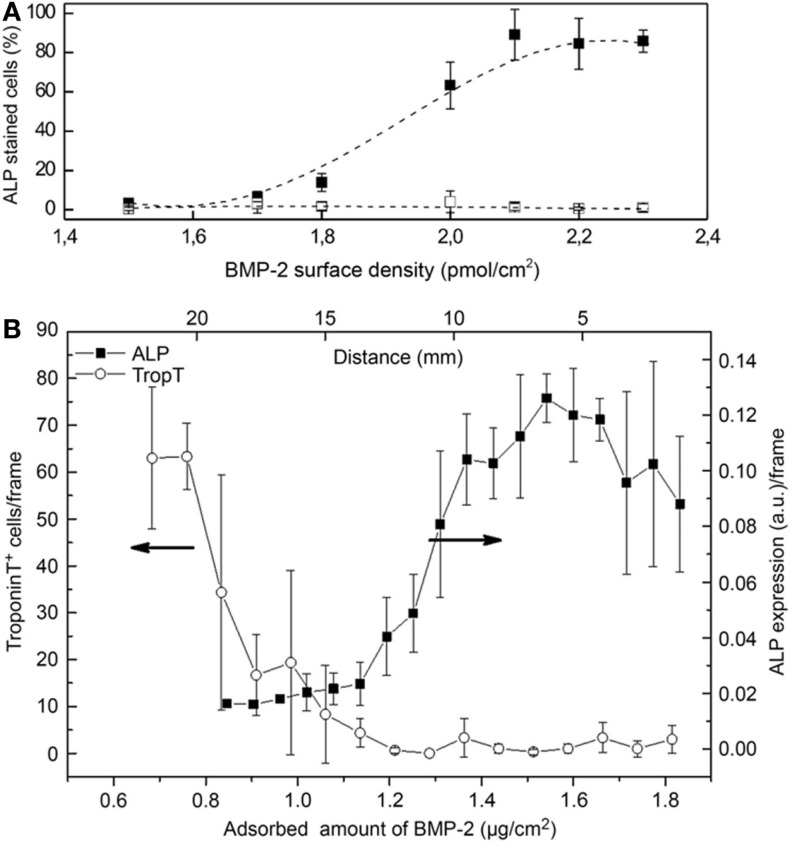
**Evolution of differentiation markers in cells cultured onto BMP-2 gradients**. **(A)** Plot of the percentage of cells in the osteogenic commitment showing ALP positive staining versus BMP-2 surface density. Non-linear effects are found below 2.1 pmol/cm^2^ and the percentage of cells in the osteogenic commitment reaches saturation from this value onward. Control experiments performed on streptavidin-modified gradients show differentiation values below 10%, independent of the BMP-2 dose. Dashed lines are an eye guide. **(B)** Differentiation of C2C12 myoblasts on BMP-2 gradients. Immuno-fluorescent imaging reveals a decrease of troponin T positive cells (undergoing myogenic differentiation) with increasing BMP-2 concentration and an increase in the ALP expression [**(A)**: Lagunas et al., 2013; **(B)**: Almodóvar et al., 2014].

In our group, we took advantage of a biotin–streptavidin gradient platform to study the concentration effects of BMP-2 on C2C12 cell differentiation (Figure 2A; Lagunas et al., 2013). We fabricated gradients of biotinylated BMP-2 bound to PMMA substrates with low slope (0.9 pmol/cm^3^) and an overall surface density ranging 1.4–2.3 pmol/cm^2^. We observed a non-linear dependence of the osterix (OSX) nuclear translocation (an osteoblast-specific transcription factor) with an abrupt increase above a threshold density of 1.7 pmol/cm^2^. A similar behavior was seen for the expression of alkaline phosphatase (ALP) enzyme, also related to osteogenic cell commitment. We attributed such non-linear behavior to the non-even distribution of the ligand (BMP-2) on the surface at the nanometer scale: BMP-2 clustered distribution synergistically enhances the probability of rebinding events by providing a large number of adjacent binding sites in the vicinity of the receptor, thereby increasing signaling activation (Lagunas et al., 2012). Such a non-linear C2C12 cell behavior in the presence of BMP-2 has been also reported in a recent work performed by Picart and co-workers (Figure 2B; Almodóvar et al., 2014). They used microfluidics to create gradients of BMP-2 and BMP-7 growth factors on layer-by-layer films composed of poly(l-lysine) and hyaluronan. The gradients have slopes of 0.58 μg/cm^3^ (22.3 pmol/cm^3^) for BMP-2 and 1.24 μg/cm^3^ (25 pmol/cm^3^) for BMP-7, and are linear over a distance of 20 mm. The effects of the gradients on the trans-differentiation capacity of the C2C12 cells to the osteogenic lineage were assessed. This platform was used then to produce gradients of both factors in a parallel or opposite fashion and the data found suggest an additive or synergistic effect between BMP-2 and BMP-7.

Also recently, surface density gradients of immobilized nerve growth factor (NGF) on plasma polymer films have been used to assess the critical growth factor density required to support neural lineage generation from mouse embryonic stem cells (Delalat et al., 2015). The authors prepared first a chemical surface gradient varying from high hydroxyl to high aldehyde group densities and then immobilized the NGF by reductive amination with the aldehyde groups. They found a critical surface density value of 52.9 ng/cm^2^ (corresponding to 1.9 pmol/cm^2^), above which cell attachment and differentiation does not increase further.

The role of molecular gradients in axonal development has been recurrently studied by means of surface-bound biomolecule gradients. Laminin gradients fabricated by microfluidic devices have been proposed to study the axon growth rate and growth direction in response to the gradient slope (Dertinger et al., 2002; Xiao et al., 2013). The axon polarization and growth response has also been addressed by gradients of covalently bound netrin-1 and brain-derived neurotrophic factor (BDNF) proteins through diffusive printing technique (Mai et al., 2009). The authors found that bound BDNF gradients caused an attractive/repulsive bidirectional response on regions of BDNF low and high densities, depending on the basal level of cyclic Adenosine MonoPhosphate in the neurons. Lang and co-workers studied the cell response to the concentration of repulsive axon guidance molecule ephrinA5 through gradients produced by microfluidic networks (Lang et al., 2008). They found that temporal, but not nasal, axons stopped at characteristic zones in the gradient, and that such stop zones were dependent on the slope of the gradient. These findings indicated that the growth cone can adjust its sensitivity during the detection of a concentration gradient of ephrinA5 and demonstrated the potential of gradients to screen cell dose response.

## Conclusion

Continuous chemical gradients covering a biologically relevant range of concentrations allow determining in an efficient way the optimal concentration of matrix-bound biomolecules for a specific cell response. Moreover, continuous chemical gradients in the examples presented showed that threshold concentrations exist for cell responses that can be related to specific ligand distributions on the surface at nanometer scale. Also synergistic effects when combining different signaling molecules in a gradient can be observed. Altogether, this review shows the potential of continuous chemical gradients for the study of ligand effects on cell behavior.

## Conflict of Interest Statement

The authors declare that the research was conducted in the absence of any commercial or financial relationships that could be construed as a potential conflict of interest.

## References

[B1] Almodóvar J. Crouzier T. Selimovic S. Boudou T. Khademhosseini A. Picart C. (2013). Gradients of physical and biochemical cues on polyelectrolyte multilayer films generated via microfluidics. Lab. Chip 13, 1562–1570. 10.1039/c3lc41407h 23440074 PMC4155072

[B2] Almodóvar J. Guillot R. Monge C. Vollaire J. Selimović S. Coll J. L. (2014). Spatial patterning of BMP-2 and BMP-7 on biopolymeric films and the guidance of muscle cell fate. Biomaterials 35, 3975–3985. 10.1016/j.biomaterials.2014.01.012 24485790 PMC4155073

[B3] Arnold M. Hirschfeld-Warneken V. C. Lohmüller T. Heil P. Blümmel J. Cavalcanti-Adam E. (2008). Induction of cell polarization and migration by a gradient of nanoscale variations in adhesive ligand spacing. Nano Lett. 8, 2063–2069. 10.1021/nl801483w 18558788 PMC3811077

[B4] Bélisle J. M. Levin L. A. Costantino S. (2012). High-content neurite development study using optically patterned substrates. PLoS One 7:e35911. 10.1371/journal.pone.0035911 22563416 PMC3338543

[B5] Deeg J. A. Louban I. Aydin D. Selhuber-Unkel C. Kessler H. Spatz J. P. (2011). Impact of local versus global ligand density on cellular adhesion. Nano Lett. 11, 1469–1476. 10.1021/nl104079r 21425841 PMC3806292

[B6] Delalat B. Mierczynska A. Ghaemi S. R. Cavallaro A. Harding F. J. Vasilev K. (2015). Materials displaying neural growth factor gradients and applications in neural differentiation of embryoid body cells. Adv. Funct. Mater. 25, 2737–2744. 10.1002/adfm.201500595

[B7] Dertinger S. K. W. Jiang X. Li Z. Murthy V. N. Whitesides G. M. (2002). Gradients of substrate-bound laminin orient axonal specification of neurons. Proc. Natl. Acad. Sci. U.S.A. 99, 12542–12547. 10.1073/pnas.192457199 12237407 PMC130496

[B8] Gurdon J. B. Bourillot P. Y. (2001). Morphogen gradient interpretation. Nature 413, 797–803. 10.1038/35101500 11677596

[B9] Harding F. J. Clements L. R. Short R. D. Thissen H. Voelcker N. H. (2012). Assessing embryonic stem cell response to surface chemistry using plasma polymer gradients. Acta Biomater. 8, 1739–1748. 10.1016/j.actbio.2012.01.034 22326974

[B10] Lagunas A. Comelles J. Martínez E. Prats-Alfonso E. Acosta G. A. Albericio F. (2012). Cell adhesion and focal contact formation on linear RGD molecular gradients: study of non-linear concentration dependence effects. Nanomedicine 8, 432–439. 10.1016/j.nano.2011.08.001 21856276

[B11] Lagunas A. Comelles J. Martínez E. Samitier J. (2010). Universal chemical gradient platforms using poly(methyl methacrylate) based on the biotin-streptavidin interaction for biological applications. Langmuir 26, 14154–14161. 10.1021/la102640w 20712344

[B12] Lagunas A. Comelles J. Oberhansl S. Hortigüela V. Martínez E. Samitier J. (2013). Continuous bone morphogenetic protein-2 gradients for concentration effect studies on C2C12 osteogenic fate. Nanomedicine 9, 694–701. 10.1016/j.nano.2012.12.002 23313904

[B13] Lang S. von Philipsborn A. C. Bernard A. Bonhoeffer F. Batsmeyer M. (2008). Growth cone response to ephrin gradients produced by microfluidic networks. Anal. Bioanal. Chem. 390, 809–816. 10.1007/s00216-007-1363-3 17557153 PMC2755754

[B14] Lee J. Choi I. Yeo W.-S. (2013). Preparation of gradient surfaces by using a simple chemical reaction and investigation of cell adhesion on a two-component gradient. Chemistry 19, 5609–5616. 10.1002/chem.201203215 23463672

[B15] Mai J. Fok L. Gao H. Zhang X. Poo M. (2009). Axon initiation and growth cone turning on bound protein gradients. J. Neurosci. 29, 7450–7458. 10.1523/JNEUROSCI.1121-09.2009 19515913 PMC6665406

[B16] Malmström J. Christensen B. Jakobsen H. P. Lovmand J. Foldbjerg R. Sorensen E. S. (2010). Large area protein patterning reveals nanoscale control of focal adhesion development. Nano Lett. 10, 686–694. 10.1021/nl903875r 20044840

[B17] Miller E. D. Fisher G. W. Weiss L. E. Walker L. M. Campbell P. G. (2006). Dose dependent cell growth in response to concentration modulated patterns of FGF-2 printed on fibrin. Biomaterials 27, 2213–2221. 10.1016/j.biomaterials.2005.10.021 16325254

[B18] Miller E. D. Phillippi J. A. Fisher G. W. Campbell P. G. Walker L. M. Weiss L. E. (2009). Inkjet printing of growth factor concentration gradients and combinatorial arrays immobilized on biologically-relevant substrates. Comb. Chem. High Throughput Screen. 12, 604–618. 10.2174/138620709788681907 19601758

[B19] Papp K. Szittner A. Prechl J. (2012). Life on a microarray: assessing live cell functions in a microarray format. Cell. Mol. Life Sci. 69, 2717–2725. 10.1007/s00018-012-0947-z 22391673 PMC11115177

[B20] Petty R. T. Li H.-W. Maduram J. H. Ismagilov R. Mrksich M. (2007). Attachment of cells to islands presenting gradients of adhesion ligands. J. Am. Chem. Soc. 129, 8966–8967. 10.1021/ja0735709 17602634 PMC2543034

[B21] Reynolds P. M. Pedersen R. H. Riehle M. O. Gadegaard N. (2012). A dual gradient assay for the parametric analysis of cell-surface interactions. Small 8, 2541–2547. 10.1002/smll.201200235 22678878

[B22] Rodríguez-Seguí S. A. Pons-Ximenez J. I. Sevilla L. Ruiz A. Colpo P. Rossi F. (2011). Quantification of protein immobilization on substrates for cellular microarray applications. J. Biomed. Mater. Res. A 98A2, 245–256. 10.1002/jbm.a.33089 21626656

[B23] Schwab E. H. Pohl T. L. M. Haraszti T. Schwaerzer G. K. Hiepen C. Spatz J. P. (2015). Nanoscale control of surface immobilized BMP-2: toward a quantitative assessment of BMP-mediated signaling events. Nano Lett. 15, 1526–1534. 10.1021/acs.nanolett.5b00315 25668064

[B24] Trappmann B. Gautrot J. E. Connelly J. T. Strange D. G. T. Li Y. Oyen M. L. (2012). Extracellular-matrix tethering regulates stem-cell fate. Nat. Mater. 11, 642–649. 10.1038/nmat3339 22635042

[B25] Wang P. Y. Clements L. R. Thissen H. Tsai W. B. Voelcker N. H. (2015). Screening rat mesenchymal stem cell attachment and differentiation on surface chemistries using plasma polymer gradients. Acta Biomater. 11, 58–67. 10.1016/j.actbio.2014.09.027 25246312

[B26] Warmflash A. Sorre B. Etoc F. Siggia E. D. Brivanlou A. H. (2014). A method to recapitulate early embryonic spatial patterning in human embryonic stem cells. Nat. Methods 11, 847–854. 10.1038/nmeth.3016 24973948 PMC4341966

[B27] Wen J. H. Vincent L. G. Fuhrmann A. Choi Y. S. Hribar K. C. Taylor-Weiner H. (2014). Interplay of matrix stiffness and protein tethering in stem cell differentiation. Nat. Mater. 13, 979–987. 10.1038/nmat4051 25108614 PMC4172528

[B28] Wittle J. D. Barton D. Alexander M. R. Short R. D. (2003). A method for the deposition of controllable chemical gradients. Chem. Commun. 2003, 1766–1767. 10.1039/b305445b

[B29] Xiao R. R. Zeng W. J. Li Y. T. Zou W. Wang L. Pei X. F. (2013). Simultaneous generation of gradients with gradually changed slope in a microfluidic device for quantifying axon response. Anal. Chem. 85, 7842–7850. 10.1021/ac4022055 23865632

[B30] Zelzer M. Majani R. Bradley J. W. Rose F. R. A. J. Davies M. C. Alexander M. R. (2008). Investigation of cell-surface interactions using chemical gradients formed from plasma polymers. Biomaterials 29, 172–184. 10.1016/j.biomaterials.2007.09.026 17949809

